# Space–Time Clustering and Climatic Risk Factors for Lumpy Skin Disease of Cattle in Uttar Pradesh, India, 2022

**DOI:** 10.1155/2024/1343156

**Published:** 2024-03-22

**Authors:** Isha Agrawal, Barkha Sharma, Csaba Varga

**Affiliations:** ^1^Department of Pathobiology, College of Veterinary Medicine, University of Illinois at Urbana–Champaign, Urbana, Illinois, USA; ^2^U.P. Pandit Deen Dayal Upadhyay Pashu Chikitsa Vigyan Vishwavidyalaya Evam Go Anusandhan Sansthan, Mathura, India; ^3^Carl R. Woese Institute for Genomic Biology, University of Illinois at Urbana–Champaign, Urbana, Illinois, USA

## Abstract

Lumpy skin disease (LSD), a transboundary infectious disease, negatively impacts cattle health and production. The first LSD outbreaks were reported in India in 2019, and since then, LSD spread to over 15 states, including Uttar Pradesh. This study evaluated LSD cases reported by veterinarians in Uttar Pradesh, India, during 2022. Using scan statistics, Poisson models that accounted for the background cattle population were constructed to identify spatial, temporal, and space–time clusters. A negative binomial regression model was built to assess the impact of temperature and humidity on the incidence rate (IR) of LSD. A total of 112,226 cases across 33 districts were reported in 2022. A purely temporal cluster with higher-than-expected LSD rates was identified between August and October 2022. Several purely spatial clusters were identified in the western part of the state. A primary space–time cluster was detected in west Utter Pradesh between August and October 2022 that overlapped with the spatial clusters. The secondary cluster occurred between September and October 2022 in the eastern part of the state. A rise in humidity (incidence rate ratio (IRR) = 1.39; 95% CI: 1.30–1.49) and temperature (IRR = 1.16; 95% CI: 1.06–1.27) increased the IR of LSD, suggesting a seasonality of the outbreaks. The results of this study can aid animal health authorities in developing effective LSD prevention, surveillance, and control strategies among cattle in India.

## 1. Introduction

Lumpy skin disease (LSD) is a transboundary infectious disease affecting ruminants, primarily cattle and water buffalo [1]. The emergence and spread of LSD are of significant concern with global implications due to its substantial impact on cattle production and trade [2]. Consequently, the World Organization for Animal Health (WOAH) has designated LSD as a notifiable animal disease [3]. The first case of LSD was reported from Zambia in 1929 [4]. Since then, it has exhibited transnational and transcontinental spread patterns, affecting numerous countries [5]. More recently, between 2019 and 2021, LSD emerged in Asian countries, including those in South Asia (Bangladesh, India, Nepal, Bhutan, Sri Lanka, and Pakistan), East Asia (China, Hong Kong), and Southeast Asia (Vietnam, Myanmar, Thailand) [6, 7].

In August 2019, India reported its first LSD case in cattle in Odisha state [8]. The disease entered India through unregulated cross-border animal movement between India and Bangladesh [6]. Since then, LSD has spread to over 15 Indian states by 2022 [9], including Uttar Pradesh (UP)—a state known for its large cattle population and substantial milk production [10]. The economic repercussions of LSD outbreaks due to livestock and production loss and additional disease management costs have been devastating for small and medium-scale cattle producers [10].

LSD is a viral disease caused by an enveloped DNA virus (lumpy skin disease virus, LSDV) of the genus Capripoxvirus of the Poxviridae family [11]. The LSD signs in cattle are fever, enlarged lymph nodes, anorexia, depression, emaciation, and the development of generalized skin nodules [12]. The production-related implications of LSD in affected animals involve reduced milk and meat production, increased rates of abortion, male infertility, and compromised hide quality [4]. The morbidity (3%–85%) and mortality (1%–40%) rates associated with LSD vary depending on the virus strain, host susceptibility, host immune response, and vector abundance in the region [4, 9]. LSD-free regions with naïve host cattle populations have reported higher morbidity and mortality than those with LSD-endemic status [13, 14]. This difference could be attributed to the acquired immunity of the host, vaccination status, and differences in farm and health management practices [15, 16].

Multiple risk factors and transmission routes of LSD have been described, including direct contact with infected cattle, transmission via semen, intrauterine transmission, or indirect transmission via fomites and aerosols [17]. Data from several studies have indicated mechanical transmission as an important route of infection, primarily by blood-sucking arthropod vectors like mosquitoes, biting flies, midges, and ticks [15, 17]. Moreover, animal movement (between infected and susceptible regions), illegal cattle importation, cattle density, and climatic factors (temperature, humidity) were described as risk factors for LSD transmission and spread [15]. Previous research has established an association between climatic factors like temperature, humidity, and precipitation and LSD outbreaks, suggesting seasonal patterns in LSD emergence [15]. These climatic risk factors might influence the density and distribution of vector populations, which could aid the spread of LSD [18].

With the emergence of transboundary infectious diseases, evaluating their spatial distribution and clustering helps to understand the host–agent–environment interplay in disease transmission and spread [19, 20]. Integrating spatial epidemiological methods in disease surveillance and monitoring is crucial for designing disease prevention and control plans [20]. Applying spatial, temporal, and space–time scan statistics allows for the identification of disease clusters where the incidence of disease exceeds expected levels and where disease-controlling measures should focus [21].

Monitoring and surveillance of LSD are essential for early disease detection, timely response, and targeted interventions to control LSD outbreaks. In the Indian context, a knowledge gap exists in studies that employ spatial epidemiological methods and regression analysis to identify LSD clusters and investigate the influence of climatic risk factors on LSD incidence. To address this knowledge gap, this study aimed to identify spatial, temporal, and space–time clusters of LSD cases in UP in 2022. The second objective was to assess the impact of climatic factors on the incidence of LSD in the cattle population of UP.

## 2. Materials and Methods

### 2.1. Study Region and Data Collection

**Figure 1:**
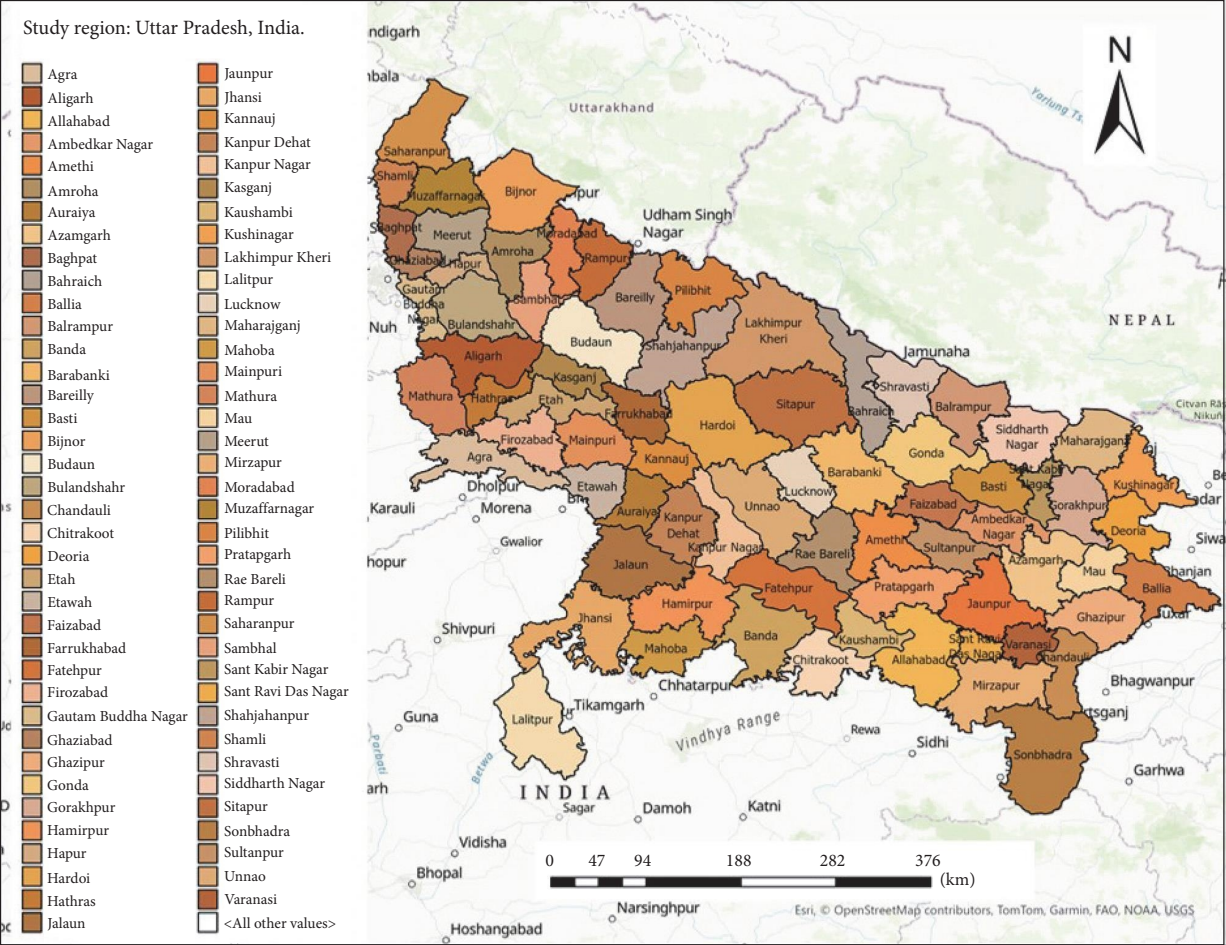
Map describing the study region in Uttar Pradesh, India.

The study evaluated the 2022 LSD outbreak in the state of UP, India, which is located in the northern part of India (26.85°N 80.91°E) with a land area of 93,023 square miles, comprising 75 districts and a total cattle population of about 18.8 million [22] (Figure 1).

This study analyzed the district-level data collected and reported by government veterinarians. The veterinary service within each district of UP is headed by a government-appointed Chief Veterinary Officer (CVO) responsible for ensuring animal health and implementing disease prevention and control plans. During an LSD outbreak, district-level data are collected and reported to the CVO of each district, who reports it to the Joint Director of Epidemiology and the Director of Disease Control at the state level.

In this study, a case was defined as a cattle exhibiting clinical signs consistent with LSD, including generalized skin nodules and enlarged lymph nodes (with or without laboratory confirmation). Confirmatory polymerase chain reaction tests were conducted on randomly collected samples (*n* = 507) from 14 affected districts. During the 2022 outbreak, active (reported by veterinarians) and passive (reported by farmers to veterinarians) approaches were used for real-time data collection. The case data were recorded as the cumulative number of cases from the affected districts in each month during the outbreak (August to November). Apart from the case data, information on the number of deaths, animals recovered, and animals vaccinated were also recorded. The data for the background cattle population was obtained from the 20th Livestock Census, 2019 (https://dahd.nic.in).

For spatial analysis, the shape file of UP was obtained from open-access data sources [23]. The shape file for the UP map was projected using the Projected Coordinate System UTM/Asia/Indian/1975UTM/Zone 47N for mapping and data visualization. Using the projected shape files, the Cartesian latitude and longitude coordinates for each district centroid were calculated in ArcGIS 10.7.1 (Environmental Systems Research Institute, Inc., Redlands, CA, USA).

To evaluate the impact of climatic risk factors (temperature, humidity, and precipitation) on LSD incidence rate (IR), district-level data on these factors were collected. The coordinates for each district centroid were used to obtain monthly data on the temperature (measured in Celsius), relative humidity (expressed as a percentage), and precipitation (measured in millimeters) for 2022 from the NASA Prediction of Worldwide Energy Resource (POWER) [24].

### 2.2. Data Analysis

#### 2.2.1. Exploratory Spatial Analysis

The IR of LSD in 2022 was calculated for each district by dividing the total number of LSD cases in a district by the total cattle population estimates. A choropleth map was constructed to illustrate the IR as the number of LSD cases per 100,000 cattle in UP using ArcGIS Pro version 3.0.3 (Environmental Systems Research Institute, Inc. (ESRI), Redlands, CA, USA) software. The natural breaks (Jenks) classification method was selected a priori and used to minimize within-class variance and maximize the between-class variance [25]. This classification was modified by adding an interval with zero IR to highlight the districts with no LSD cases.

#### 2.2.2. Scan Statistics

To detect areas and periods with higher-than-expected LSD cases, using the SaTScan software version 9.6., we constructed discrete Poisson models [25]. A Poisson model follows the assumption that the number of LSD cases in each district follows a Poisson distribution with a known underlying population at risk [26]. We employed the scan statistic to identify discrete purely spatial, purely temporal, and space–time clusters of LSD cases. For the spatial scan statistic, we used a circular scanning window to detect large, compact clusters, setting the maximum cluster size to include 50% of the study population.

For the temporal analysis, the smallest unit was set to 1 month, considering the incubation period of 1–4 weeks of LSD in natural infections [4], and the maximum cluster size was set to 6 months (50% of the study period). For the space–time analysis, a cylindrical scanning window with a circular spatial base and the height corresponding to time was used, and the maximum cluster size was set to include 50% of the population and study period [25]. For space–time analysis, the scanning window moves simultaneously in space and time, comparing the rate of LSD cases inside the scanning window to the rate of cases outside the scanning window [25]. LSD is a transboundary infectious disease mainly transmitted through mechanical vectors that could be widespread; therefore, including up to 50% of the study population and up to 50% of the study period would facilitate the detection of large primary and secondary clusters. The statistical significance of the clusters was determined at *p*-value ≤ 0.05 and validated through 999 Monte Carlo simulations [25].

For purely spatial and space–time scan statistics, if the LSD IR was significantly (*p*-value ≤ 0.05) higher inside the window compared to outside, a high-rate cluster (primary cluster, with the highest relative risk, RR) was identified. In addition to the primary cluster, we reported one or more secondary clusters if they did not geographically overlap with the primary cluster and were statistically significant.

The RR of LSD in districts within the significant spatial and space–time clusters was calculated and illustrated in maps to circumvent the assumptions that the LSD RR within a significant cluster is uniform [27].

The statistically significant spatial and space–time clusters were illustrated in ArcGIS Pro version 3.0.3 (Environmental Systems Research Institute, Inc. (ESRI), Redlands, CA, USA).

### 2.3. Assessment of Climatic Risk Factors

#### 2.3.1. Descriptive Analysis

Within UP, nine agroclimatic zones (number of districts in that zone, *n*) have been identified: Terai (*n* = 9), western plains (*n* = 8), central western plains (*n* = 7), semiarid southwestern plains (*n* = 8), central plains (*n* = 13), Bundelkhand (*n* = 7), northeastern plains (*n* = 11), eastern plains (*n* = 15), and Vindhyachal (*n* = 3) [28]. To assess the role of climatic risk factors on LSD incidence, group line graphs of LSD IR with monthly variation in temperature, relative humidity, and precipitation of one randomly selected district from each of the nine agroclimatic zones were plotted using R Studio (Version 1.4.1106 2009-2021 RStudio, PBC). This relationship was further evaluated for association using regression analysis.

#### 2.3.2. Regression Analysis

A Poisson regression model [29] was constructed using the backward selection technique evaluating the association between the district-level LSD cases (outcome) accounting for the background cattle population in each district (exposure) and climatic risk factors (temperature, precipitation, and relative humidity: predictors). The Poisson model was tested for over-dispersion. As the model was over-dispersed (*χ*^2^ = 1,775,283, *p*  < 0.001), signifying a lack of fit, a negative binomial regression model [30] was chosen. Precipitation was excluded from the final model during the backward selection due to a nonsignificant *p*-value (0.619). Incidence rate ratios (IRRs), 95% Confidence Intervals, and *p*-value were reported for each predictor variable. The regression analysis was done using STATA Intercooled software (Version 17, Stata Corporation, College Station, TX).

## 3. Results and Discussion

### 3.1. Exploratory Spatial Analysis

**Figure 2:**
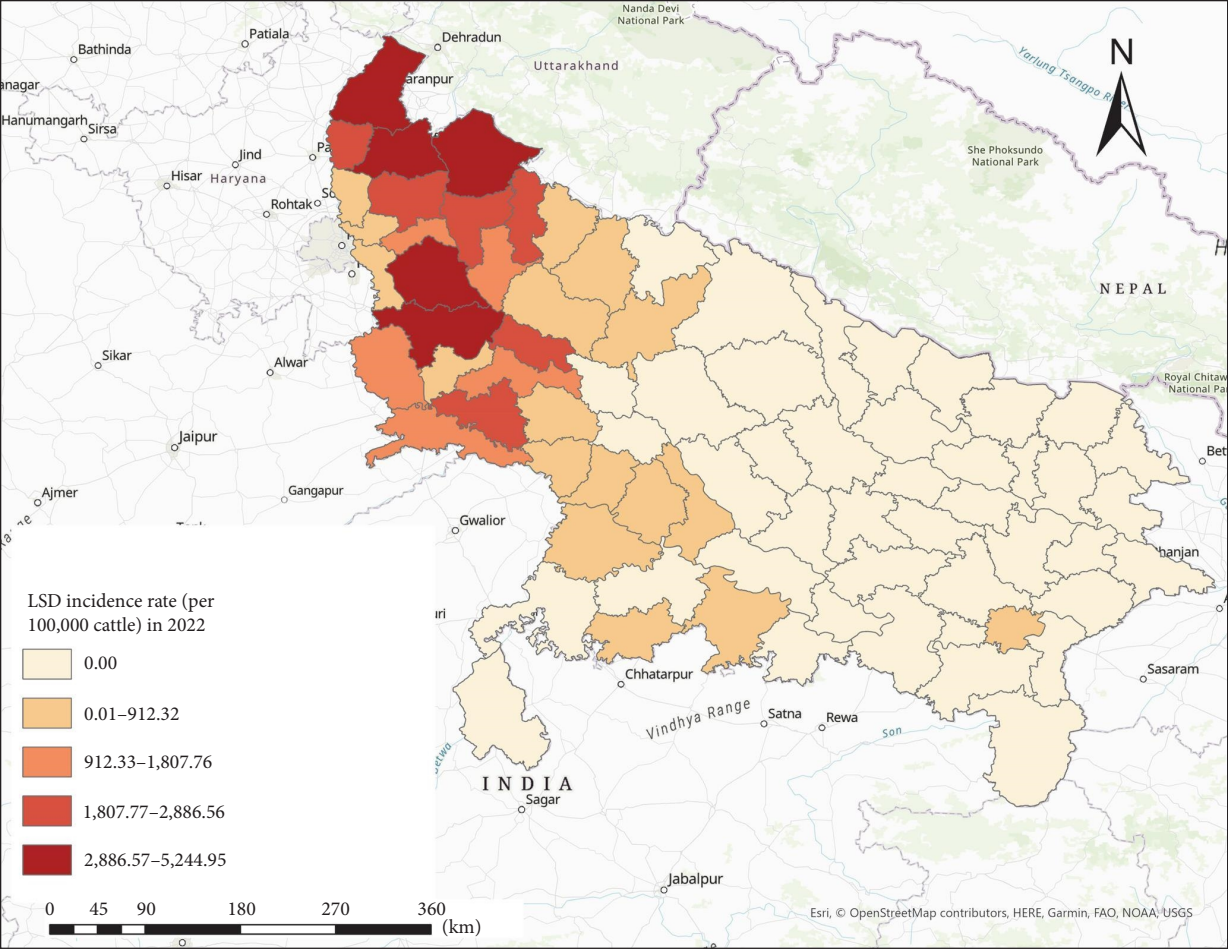
Incidence rate of lumpy skin disease per 100,000 cattle in Uttar Pradesh, India, 2022.

Out of the 75 districts in UP, 112,226 LSD cases were reported from 33 districts during the 2022 outbreak. The outbreak spanned over 4 months, from August to November, with no additional cases reported in the other 8 months. At the beginning of the outbreak in August, 18,569 LSD cases were reported from 22 districts, followed by a rise to an additional 32,871 cases in September from 29 districts in UP. The peak in the outbreak was reported in October, with 56,300 LSD cases across 32 districts. Thereafter, a dip in LSD cases (*n* = 4,486) and affected districts (*n* = 25) was observed in November, with no case reported in December 2022. The highest IR per 100,000 cattle was observed in the western part of the state in Saharanpur, Muzaffarnagar, Bijnor, Aligarh, and Bulandshahr districts. The Eastern (except Varanasi) and Central parts of UP remained unaffected (Figure 2).

### 3.2. Scan Statistics

#### 3.2.1. Purely Spatial Cluster Analysis

**Figure 3:**
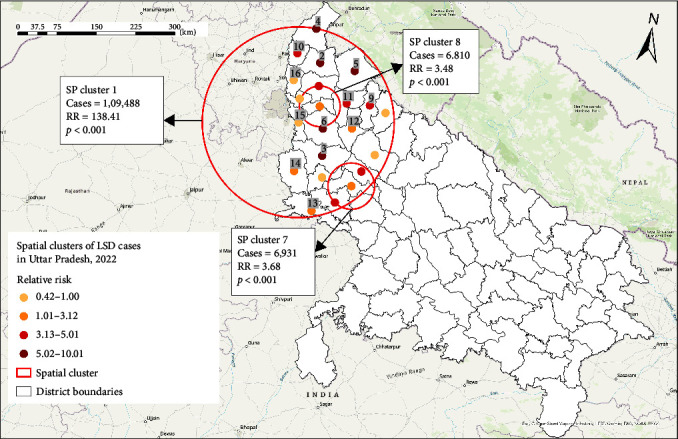
High-rate spatial clusters of LSD cases in Uttar Pradesh, 2022, identified by scan statistic. Retrospective analysis, scanning for clusters with high rates, using a discrete Poisson model.

Sixteen purely spatial high-rate LSD clusters were identified. The primary cluster (SP1, cluster radius: 173.19 km) with the highest RR of 138.41, included 23 districts (Gautam Buddha Nagar, Ghaziabad, Bulandshahr, Hapur, Aligarh, Meerut, Baghpat, Mathura, Amroha, Sambhal, Hathras, Muzaffarnagar, Shamli, Moradabad, Bijnor, Kasganj, Etah, Budaun, Rampur, Firozabad, Agra, and Saharanpur) located in the western part of UP. Additional 15 secondary clusters were identified, encompassing one, two, or three districts with an RR ranging between 1.2 and 10.02 (Table 1 and Figure 3).

#### 3.2.2. Temporal Cluster Analysis

One purely temporal cluster of higher-than-expected LSD cases (*n* = 107,740; RR = 71.72) was identified between August and October 2022 (Table 2).

#### 3.2.3. Space–Time Cluster Analysis

**Figure 4:**
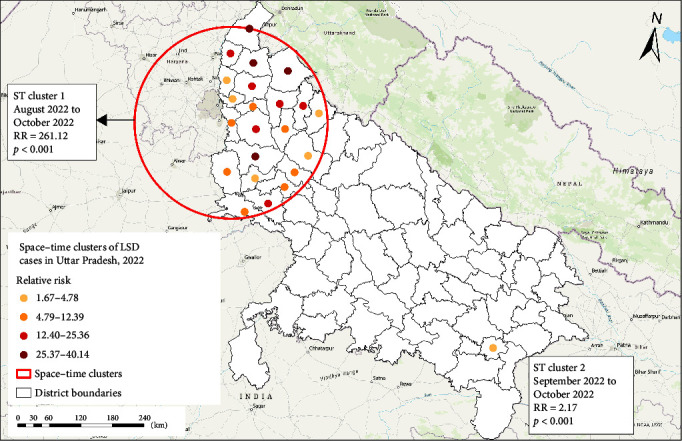
High-rate space–time clusters of LSD cases in Uttar Pradesh in 2022, detected by the scan statistic. Retrospective analysis, scanning for clusters with high rates, using a discrete Poisson model.

The space–time discrete Poisson model identified two LSD clusters (Table 2 and Figure 4).

The primary cluster (ST1), which occurred between August and October 2022, encompassed 22 districts (Agra, Aligarh, Amroha, Budaun, Baghpat, Bijnor, Bulandshahr, Etah, Firozabad, Gautam Buddha Nagar, Ghaziabad, Hapur, Hathras, Kasganj, Mathura, Meerut, Moradabad, Muzaffarnagar, Rampur, Sambhal, Saharanpur, and Shamli) located in the western parts of UP (Figure 4). The space–time cluster geographically overlapped with the spatial cluster. All districts included in the space–time primary cluster (ST1) had RR > 1 (range: 1.66–40.14). Within the cluster, the highest number of LSD cases were reported from district Bijnor (*n* = 14,960) and Muzaffarnagar (*n* = 14,761). The second cluster (ST2) was restricted to the district Varanasi, located in the eastern part of UP, and occurred between September and October 2022 with an RR of 2.16 (Table 2).

### 3.3. Climatic Risk Factors

**Figure 5:**
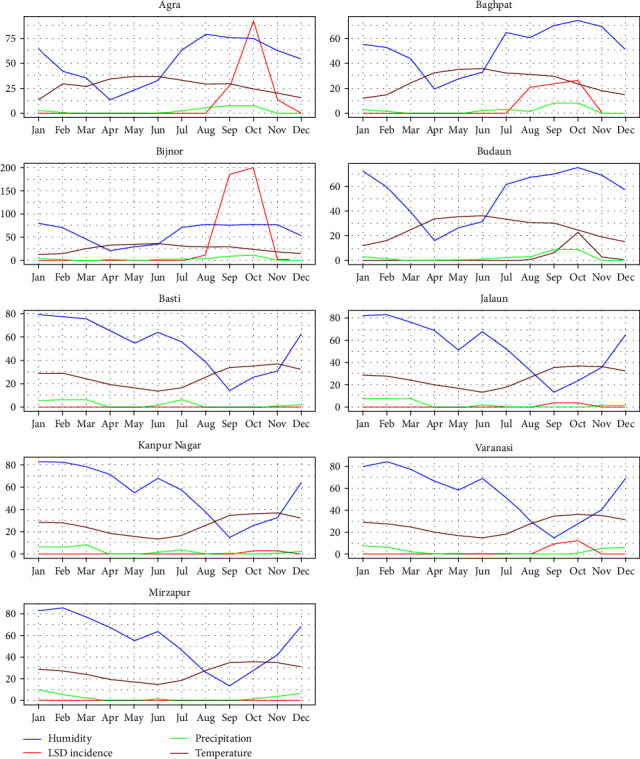
Monthly trend in LSD incidence rate (per 10,000 cattle) and climatic factors in nine different agroclimatic zones in Uttar Pradesh in 2022. Semiarid southwestern plains (Agra), western plains (Baghpat), Terai (Bijnor), central western plains (Budaun), northeastern plains (Basti), Bundelkhand (Jalaun), central plains (Kanpur Nagar), eastern plains (Varanasi), and Vindhyachal (Mirzapur).

Figure 5 shows monthly temperature, precipitation, and relative humidity variation in randomly selected districts from each of the nine agroclimatic zones in UP.

A seasonal trend in the distribution of LSD cases (IR per 10,000 cattle) in the nine agroclimatic zones was evident in Figure 3. A peak in LSD cases was observed between August and October, particularly in the districts in the western region of UP (Agra, Baghpat, Bijnor, Badaun). This peak in IRs overlapped with the rise in temperature, precipitation, and relative humidity (Figure 5). A dip in relative humidity coincided with near-zero or low IRs of LSD cases in the central and eastern agroclimatic zones of UP.

### 3.4. Negative Binomial Regression

The multivariable negative binomial regression analysis indicated that the IRR of LSD would be expected to increase by a factor of 1.16 (95% CI: 1.06–1.27) with a unit increase in the temperature and by a factor of 1.39 (95% CI: 1.30–1.49) with a unit increase in the relative humidity.

## 4. Discussions

This study analyzed LSD outbreak data collected by government veterinarians in UP, India, by using spatial epidemiological approaches to assess the distribution, clustering, and risk factors of LSD cases across 75 districts in UP, India, during the 2022 LSD outbreak. Purely spatial, temporal, and space–time scan statistics were conducted to identify locations and periods with higher-than-expected LSD rates. Additionally, data on climatic risk factors, such as temperature, relative humidity, and precipitation, were collected for each district and month to evaluate their impact on the IR of LSD using descriptive and regression analysis.

The scan statistical methods [25, 31] have been commonly used by previous studies to detect a local excess of cases and test if these occurrences were random or clustered by location and/or time [25, 27, 32, 33]. A high-rate spatial, temporal, and space–time cluster in a specific location and/or period provides insights into disease epidemiology and helps health authorities focus on areas with high infection rates. This aids in the directed planning of the prevention and control measures to mitigate further disease transmission and spread [21, 31]. To avoid misclassification of districts within significant spatial and space–time clusters, we reported the RR of LSD occurrence for each district within a significant cluster to be able to differentiate between districts with low and high RRs (Figures 3 and 4) [21], which is valuable information when implementing disease mitigation measures for a region during an LSD outbreak.

The temporal cluster in the study revealed higher-than-expected LSD infection rates between August and September, which represents the months of the rainy season in UP [34]. This season provides favorable environmental conditions for the emergence of the LSD vector populations that partly could explain the increase in LSD in local cattle populations in UP. Several previous studies have shown the role of mechanical vectors, such as Aedes, Culex, Anopheles mosquitoes, stable flies, and other biting arthropods, in the transmission of LSD [15, 17, 35–38], and these vectors are abundant in UP [39–42], suggesting a connection between vector abundance and increase in LSD cases observed during these months.

The spatial cluster analysis revealed one primary cluster that involved 23 districts in the western part of UP. Interestingly, the space–time cluster analysis identified a primary cluster involving 22 districts in the western UP between August and October that overlapped with the purely spatial cluster. The high LSD rates in the western part of the state might be partly attributed to the unrestricted interstate movement of LSD-infected cattle from neighboring states, as media and government reports described LSD outbreaks in the previous months (May–July) in states (Rajasthan, Uttarakhand, Haryana) sharing a border with western districts of UP [8, 9, 43]. The secondary high-rate space–time cluster was restricted to only one district (Varanasi) in east UP, occurring from August to October, which could be attributed to a long-distance LSD transmission through mechanical vectors, animal movement, or a higher susceptibility of the cattle population due to lack of previous exposure or vaccination [17]. Moreover, the higher LSD incidence could also be due to higher transmission through mechanical vectors as the higher-than-expected LSD rate periods in both LSD space–time clusters represent the mid-to-late rainy season in UP with warm and humid conditions that favor the increase in the vector population. Similar seasonality in LSD outbreaks has been reported by previous studies conducted in the Middle East [18], Ethiopia [45], Egypt [46], Uganda [47], and Thailand [14].

To further explore the seasonality pattern, we evaluated associations between LSD incidence and climatic factors. First, each month's average temperature, relative humidity, and precipitation in each district were collected, and descriptive graphs were designed to visually assess underlying LSD patterns with variations in the climatic risk factors. Next, a negative binomial regression model was constructed to establish associations between LSD incidence as the outcome and temperature and relative humidity as predictor variables. The results suggested a positive effect of temperature and relative humidity on the IR of LSD. This finding is consistent with the pattern observed in the descriptive analysis and previous studies [14, 18, 44–46]. This finding could be explained by the favorable effect temperature and humidity (warm and humid conditions) have on the increase in the vector population [15, 44, 45]. Besides climatic factors, indirect (through fomites) and direct (infected to susceptible cattle) [17] transmission of the virus should also be considered, as the LSD virus can survive for over 33 days in necrotic nodules of the skin of infected cattle [5].

Discussing this study's limitations is imperative before interpreting its findings. The study was conducted on data collected actively and passively by veterinarians deployed in each district in UP, introducing a reporting bias in the LSD case count [47]. Moreover, after the confirmatory tests in a region, additional cases were identified and reported based on the clinical signs alone. However, reporting LSD cases based on clinical signs during a large outbreak is common due to time, cost, and resource limitations, and conducting confirmatory tests on all suspect animals is not feasible [48–50]. A limitation of the Poisson-based spatial scan statistic is that locations with high RRs absorb insignificant neighbors with nonelevated risks into the significant cluster. To overcome this issue, the RRs of all locations within the significant cluster were presented in the cluster maps.

The generalizability of the study results to other states in India or other countries should be taken with caution as this study was conducted only in UP, which may differ in the geographic features, cattle population, production system, climatic conditions, vector population, animal movements, and other prevention and control measures imposed by various state governments. However, a similar spatial analytical methodology could be applied to analyze LSD outbreak data in other states to get more precise information on LSD spread and clustering in those areas.

## 5. Conclusions

This study demonstrated the utility of the spatial, temporal, and space–time scan statistics in identifying locations and periods with higher-than-expected LSD rates in cattle populations. Between August and October 2022, a large cluster with a higher-than-expected LSD rate was identified in the western part of UP. Future studies should focus on this area to identify local factors, including interstate movement of LSD-infected cattle, local vaccination coverage, and climatic factors that might impact the distribution and spread of LSD among cattle. High temperature and humidity impacted the incidence of LSD cases, suggesting that the vector population abundance in warmer months might play a role in LSD transmission. The study methodology can be used by animal health authorities to facilitate rapid disease response and develop effective disease prevention and mitigation measures.

## Figures and Tables

**Table 1:** Description of significant spatial clusters during the 2022 LSD outbreak in Uttar Pradesh, India.

Spatial clusters	Number of districts (name)	Radius (km)	Observed cases (*O*)	Expected cases (*E*)	*O*/*E*	Relative risk (RR)	Likelihood ratio	*p*-Value
SP1	23	173.19	109,488	25,155.39	4.35	138.41	151,556.5	<0.001
SP2	1: Muaffarnagar	0	14,878	1,718.38	8.66	9.83	19,771.2	<0.001
SP3	1: Aligarh	0	14,962	1,836.82	8.15	9.24	19,070.44	<0.001
SP4	1: Saharanpur	0	13,978	1,572.52	8.89	10.01	18,856.71	<0.001
SP5	1: Bijnor	0	14,978	2,232.11	6.71	7.59	16,535.53	<0.001
SP6	1: Bulandshahr	0	11,060	1,795.66	6.16	6.72	14,242.29	<0.001
SP7	3: Etah, Kasganj, Firozabad	42.23	6,931	1,971.74	3.52	3.68	3,866.8	<0.001
SP8	2: Hapur, Meerut	38.95	6,810	2,045.71	3.33	3.48	3,530.2	<0.001
SP9	1: Moradabad	0	4,213	1,044	4.04	4.15	2,754.2	<0.001
SP10	1: Shamli	0	3,403	695.62	4.89	5.01	2,728.38	<0.001
SP11	1: Amroha	205.14	4,528	1,278.34	3.54	3.65	2,525.03	<0.001
SP12	1: Sambhal	0	3,162	1,032.08	3.06	3.12	1,430.87	<0.001
SP13	1: Agra	0	3,746	1,668.60	2.24	2.29	971.65	<0.001
SP14	1: Mathura	0	2,922	1,264.11	2.31	2.35	802.91	<0.001
SP15	1: G B Nagar	0	829	536.16	1.55	1.55	68.81	<0.001
SP16	1: Baghpat	0	1,029	861.06	1.2	1.2	15.53	<0.001

**Table 2:** Description of the significant temporal and space–time clusters in the 2022 LSD outbreak in Uttar Pradesh, India.

Cluster type	Name	Number of districts (name)	Radius (km)	Time frame	Observed cases (*O*)	Expected cases (*E*)	*O*/*E*	Relative risk (RR)	Likelihood ratio	*p*-Value
Temporal	T1	All	NA	Aug 2022–Oct 2022	107,740	28,287.1	3.81	71.72	130,942.3	0.001
Space–time	ST1	22	173.19	Aug 2022–Oct 2022	105,480	6,340.54	16.64	261.12	277,988.6	<0.001
	ST2	1 (Varanasi)	0	Sept 2022–Oct 2022	655	302.59	2.16	2.17	153.9	<0.001

## Data Availability

The data used to support the findings of this study are included in the article.
